# Dried blood spot versus venous blood sampling for phenylalanine and tyrosine

**DOI:** 10.1186/s13023-020-1343-7

**Published:** 2020-04-03

**Authors:** Kimber van Vliet, Wiggert G. van Ginkel, Esther van Dam, Pim de Blaauw, Martijn Koehorst, Hermi A. Kingma, Francjan J. van Spronsen, M. Rebecca Heiner-Fokkema

**Affiliations:** 1 Division of Metabolic Diseases, University of Groningen, University Medical Center Groningen, Beatrix Children’s Hospital, Hanzeplein 1, 9713 GZ Groningen, The Netherlands; 2 Department of Laboratory Medicine, Laboratory of Metabolic Diseases, University of Groningen, University Medical Center Groningen, Hanzeplein 1, 9713 GZ Groningen, P.O. Box 30.001, 9700 RB, The Netherlands

**Keywords:** Phenylalanine, Tyrosine, Dried blood spots, Lithium heparin plasma, EDTA plasma, Tandem mass spectrometry

## Abstract

**Background:**

This study investigated the agreement between various dried blood spot (DBS) and venous blood sample measurements of phenylalanine and tyrosine concentrations in Phenylketonuria (PKU) and Tyrosinemia type 1 (TT1) patients.

**Study design:**

Phenylalanine and tyrosine concentrations were studied in 45 PKU/TT1 patients in plasma from venous blood in lithium heparin (LH) and EDTA tubes; venous blood from LH and EDTA tubes on a DBS card; venous blood directly on a DBS card; and capillary blood on a DBS card. Plasma was analyzed with an amino acid analyzer and DBS were analyzed with liquid chromatography-mass spectrometry. Agreement between different methods was assessed using Passing and Bablok fit and Bland Altman analyses.

**Results:**

In general, phenylalanine concentrations in LH plasma were comparable to capillary DBS, whereas tyrosine concentrations were slightly higher in LH plasma (constant bias of 6.4 μmol/L). However, in the low phenylalanine range, most samples had higher phenylalanine concentrations in DBS compared to LH plasma. Remarkably, phenylalanine and tyrosine in EDTA plasma were higher compared to all other samples (slopes ranging from 7 to 12%). No differences were observed when comparing capillary DBS to other DBS.

**Conclusions:**

Overall agreement between plasma and DBS is good. However, bias is specimen- (LH vs EDTA), and possibly concentration- (low phenylalanine) dependent. Because of the overall good agreement, we recommend the use of a DBS-plasma correction factor for DBS measurement. Each laboratory should determine their own factor dependent on filter card type, extraction and calibration protocols taking the LH plasma values as gold standard.

## Background

To improve the outcome in patients with phenylketonuria (PKU, OMIM #261600), frequent monitoring of blood phenylalanine (Phe) concentrations is necessary [1, 2]. Measurement of tyrosine (Tyr) concentrations can be important as well in PKU, as Tyr and Phe/Tyr ratios are found to be related to executive cognitive functioning [3]. In Tyrosinemia type 1 (TT1, OMIM #276700), attention usually focuses on monitoring Tyr concentrations [4]. Some recent theoretical and clinical studies suggested however that Phe concentrations could also be important. Low Phe concentrations have been related to impaired growth, skin problems and neurological deficits [5–8].

Monitoring Phe and Tyr concentrations is nowadays increasingly done using dried blood spots (DBS). The validity of the DBS for monitoring Phe is of ongoing debate [9–12]. A pilot study in the Netherlands for example showed high variability between laboratories in DBS Phe concentrations. Moreover, differences between lithium heparin (LH) anticoagulated plasma and DBS Phe concentrations among the participating laboratories varied considerably (Coene et al. personal communications). This could, in part, be caused by the different analytical methods used. On the other hand, differences between DBS and venous blood might especially be dependent on the applied calibration method and corresponding correction factors.

Independent of the specific cause, the large inter-laboratory variability in Phe concentrations has important implications for the patients treated in the different centers, and patient monitoring when using (inter)national target values. There are few reports on the possible differences between Tyr concentrations in DBS and venous plasma samples so far [10, 12], although it is likely that the same uncertainties as with the measurement of Phe exist.

The objective of this study was to investigate the agreement between Phe and Tyr concentrations as measured in different types of blood sampling in our laboratory. For this, we compared lithium heparin- and EDTA-plasma with capillary DBS. Additionally, we investigated the agreement between DBS spotted from capillary or different venous blood samples.

## Methods

### Subjects

In total, 40 PKU patients (18 male) and 5 TT1 patients (4 male) were included in this study (mean age ± SD: 18 years ±12.5; range 0–48 years). Five PKU and 3 TT1 patients were included multiple times at different time points to enhance the sample size (especially samples with low Phe and high Tyr concentrations). All PKU patients were treated with a Phe restricted diet and/or tetrahydrobiopterin. All TT1 patients were treated with 2-(2-nitro-4-trifluoromethylbenzoyl)-1,3-cyclohexanedione (NTBC), dietary restriction of Phe and Tyr, and Phe supplementation in case of otherwise very low plasma Phe concentrations (< 30 μmol/L) [13]. The need for formal ethical review was waived by the local ethics committee, since we made use of blood that was drawn regularly during outpatient visits. The study design was in accordance with the current revision of the Helsinki Declaration. All PKU and TT1 caregivers or patients gave written informed consent for this study. Children gave assent if age and understanding was appropriate according to ethics guidelines.

### Study design

In total, 53 measuring points in the outpatient clinic were studied. For this study, the following six ways of blood sampling were compared:

1. Plasma from venous blood sampling in regular lithium heparin (LH) tubes;
2. Plasma from venous blood sampling in K_2_-EDTA (EDTA) tubes;
3. Venous blood sampling from a LH tube on a DBS card;
4. Venous blood sampling from an EDTA tube on a DBS card;
5. Venous blood directly on a DBS card;
6. Capillary blood (by finger puncture) on a DBS card.

Venous blood was taken by venipuncture with a butterfly needle and collected in LH tubes (which is the regular tube in the UMC Groningen for Phe and Tyr analysis), EDTA tubes, and sterile syringes. Blood spots were made using blood from the syringe, and by taking a drop of LH- and EDTA-anti-coagulated blood, corresponding to approximately 40 μl, from the tubes and applying this on blood spot cards. In addition, blood spots were made by collecting one drop of capillary blood on a filter card by finger puncture (as is done at home). All blood samples from the patient were taken at the same time at the outpatient clinic to ensure minimal differences between the samples due to diurnal variation in concentrations [13–16]. Blood spot cards were obtained from Sartorius (TFN Grade 179 g/m^2^, Sartorius Stedim, Göttingen, Germany).

### Laboratory analyses

All samples were analyzed in the Laboratory of Metabolic Disease of the UMC Groningen. The laboratory is ISO15189 accredited (M078). Blood applied on filter cards was dried for at least 3 h at room temperature. DBS cards were stored at room temperature for 0–6 days prior to analyses. Analyzed data were corrected using a DBS-plasma correction factor as is practice in our hospital. The correction factor was determined by comparing concentrations of Phe and Tyr in DBS and plasma, prepared from blood samples spiked with Phe and Tyr (0–100–250-500-750-1000-1400 μmol/l). Phe and Tyr concentrations in DBS were measured with LC-MS/MS, using a calibration curve in 0.1 N HCl. Phe and Tyr concentrations in plasma were analyzed with Biochrom. Details of the methods are described in the Supplemental Materials. We then estimated the DBS-plasma correction factor using a Passing and Bablok regression analysis. In this analysis, Phe or Tyr concentrations in plasma were plotted on the x-axis, and DBS concentrations were plotted on the y-axis. The slope of the regression analyses, corresponding to 2.4 in our methods, was applied as DBS-plasma correction factor. We applied the DBS-plasma correction factor by adjusting the amount of calibration solution used in our LC-MS/MS method. The correction factor was subsequently verified using plasma and DBS samples prepared from blood samples of patients. A more detailed description of the determination of the correction factor is presented in the Supplemental Materials.

Blood collected in tubes was centrifuged within 1 h after sampling to obtain plasma. EDTA samples were centrifuged after 1–2 h, with a maximum of 4 h after sampling due to the fact that these samples were first analyzed for hemocytometric parameters for regular patient care. This period does not affect the Phe and Tyr concentrations in these tubes to a large extent; i.e. + 4.7% for Phe and no bias for Tyr [17]. Plasma was stored at − 20 °C for 1–5 days prior to analyses. Plasma concentrations were determined using a commercial method based on ion exchange chromatography with post column derivatization with Ninhydrin on a Biochrom 30+ or Biochrom 30 analyser (Pharmacia Biotech, Cambridge, UK) (for sample preparation, we refer to van Vliet et al. [18]). A physiological amino acid calibration standard was used for calibration (Sigma-Aldrich, Darmstadt, Germany). External quality control samples (ERNDIM) showed excellent accuracy, precision and recovery. The method was linear up to at least 1750 μM. Limits of quantification (LOQ) were determined based on the CV > 20% criterion and were 0.5 μM for Phe and 2.0 μM for Tyr. Variation coefficients of the internal quality control samples during the study period are shown in the Supplemental materials. DBS Phe and Tyr concentrations were determined using liquid chromatography tandem mass spectrometry (LC-MS/MS) analysis. For more details on this method we refer to the Supplemental materials.

### Statistical analyses

To compare Phe and Tyr concentrations in different sampling methods, Passing and Bablok regression analyses were performed, as recommended by the Clinical and Laboratory Standards Institute [19]. The Passing and Bablok regression analysis is a linear regression procedure without assumptions regarding the distribution of samples and measurement errors, and is less sensitive to outliers. The EP09-A3 protocol recommends analyzing a minimum of 40 samples [19, 20]. The intercept (representing constant bias) and slope (representing proportional bias) are presented as estimates and should ideally not be different from 0 (intercept) and 1 (slope), at *p* < 0.05. In addition, Bland Altman tests for relative differences were performed to visualize the relation between bias and concentration. The relative differences are expressed as percentages of the mean concentration. All statistical analyses were performed using IBM SPSS Statistics 23rd version and Analyse-it for Microsoft Excel 4.18.6 (Analyse-it Software, Ltd).

## Results

**Fig. 1:**
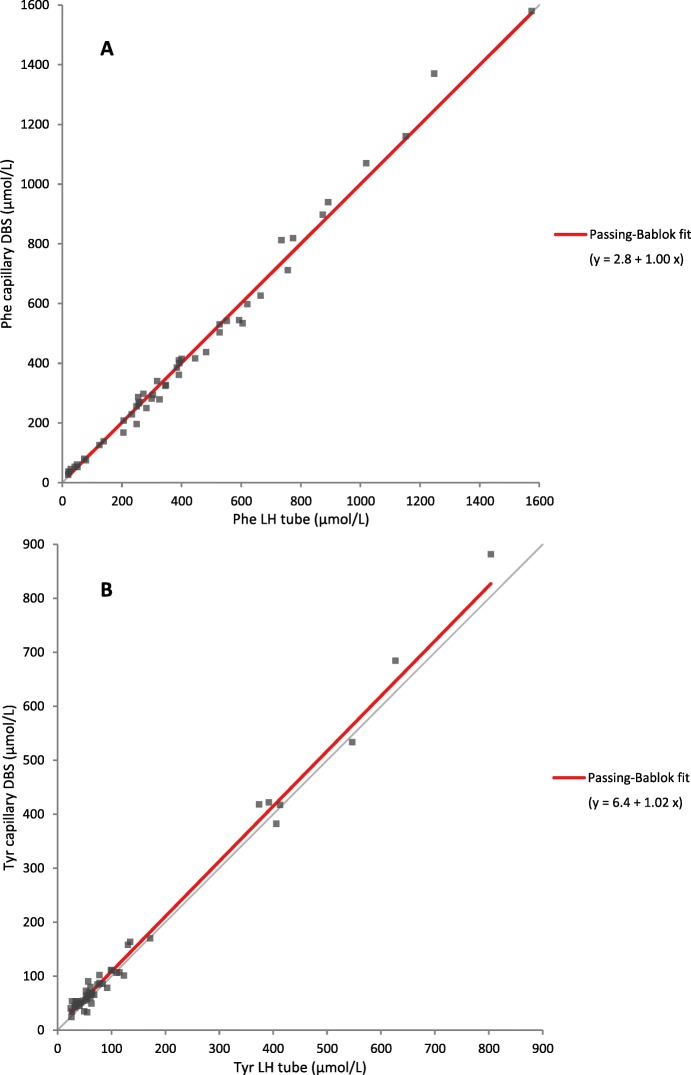
Results on Passing and Bablok fit analyses comparing **a**) phenylalanine concentrations from lithium heparin (LH) plasma and capillary dried blood spots (DBS) and **b**) tyrosine concentrations from LH plasma and capillary DBS

Table 1 shows the results of the Passing and Bablok regression analyses for the comparison of the Phe and Tyr concentrations of LH- and EDTA-anti-coagulated plasma, and capillary DBS and their respective correlation coefficients. Only the significant and the most notable results will be addressed further. In total, 6.9% of our data was missing (15 EDTA plasma samples, 3 capillary DBS, and 4 EDTA DBS). As can be observed in Table 1, the Passing and Bablok regression analyses of the comparison of LH plasma and capillary DBS for Phe showed no differences. The regression analyses for Tyr showed a higher intercept but a normal slope. These Passing and Bablok regression analyses of the post-correction data are also presented in Fig. 1.

**Table 1:** Results on Passing-and-Bablok-fit analyses for phenylalanine and tyrosine concentrations in LH plasma, EDTA plasma, and capillary DBS

**Phenylalanine**^a^		**Lithium heparin (LH) plasma**			**EDTA plasma**			**Capillary blood spot**		
	*Median (range)*	*intercept*	*slope*	*R*	*intercept*	*slope*	*R*	*intercept*	*slope*	*R*
**LH plasma**	326 (20–1574)									
**EDTA plasma**	321 (23–1707)	−2.1	1.10*	0.991						
**Capillary DBS**	325.5 (26.8–1580)	2.8	1.00	0.993	9.7	0.92*	0.978			
**Venous DBS**	309 (25.9–1490)	7.5	0.98	0.990	6.1	0.93*	0.981	0.7	1.01	0.984
**LH DBS**	297 (23.5–1610)	6.5	0.96*	0.996	4.7	0.88*	0.985	−0.7	0.98	0.991
**EDTA DBS**	311 (26.4–1550)	6.3	0.96*	0.995	5.9	0.89*	0.984	−0.6	1.00	0.992
**Tyrosine**^a^		**LH plasma**			**EDTA plasma**			**Capillary blood spot**		
	*Median (range)*	*intercept*	*slope*	*R*	*intercept*	*slope*	*R*	*intercept*	*slope*	*R*
**LH plasma**	61 (25–804)									
**EDTA plasma**	72 (29–912)	0.0	1.12*	0.982						
**Capillary DBS**	68.25 (24.2–881)	6.4*	1.02	0.930	6.8*	0.93*	0.903			
**Venous DBS**	67 (26.8–871)	3.3	1.04	0.934	4.6	0.92*	0.934	−0.9	0.99	0.934
**LH DBS**	64.1 (23.1–845)	1.8	1.05	0.951	−0.1	0.96	0.937	−4.1	1.01	0.964
**EDTA DBS**	70.9 (24.1–793)	4.5	0.98	0.959	4.7	0.91	0.934	−1.3	0.97	0.959

^a^The methods in the upper row (x) were compared to parameters in the left column (y), where y = intercept + slope * x. Results with an asterix represent significant differences with *p* < 0.05 (intercept significantly different from 0; slope significantly different from 1)

**Fig. 2:**
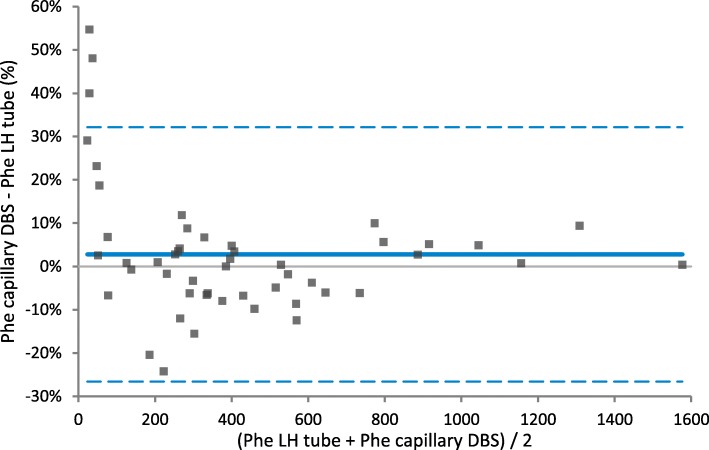
Results on Bland Altman analyses for phenylalanine concentrations measured in lithium heparin (LH) plasma compared to capillary dried blood spots (DBS)

Phe and Tyr concentrations in LH plasma were higher compared to LH DBS and EDTA DBS for Phe (4% for both), and (slightly) higher compared to capillary DBS for Tyr (intercept: 6.4 μmol/L). No differences could be demonstrated between LH plasma and other DBS. The Bland Altman difference plot (Fig. 2) revealed that there might be differences between LH plasma and capillary DBS for Phe concentrations of approximately < 50 μmol/L, i.e. low for PKU. In this Phe range, Phe concentrations in LH plasma tended to be lower than Phe concentrations measured in capillary DBS. This was mainly due to three outliers, deriving from TT1 patients with low Phe concentrations (see Fig. 2).

No differences in Phe and Tyr concentrations were observed when comparing all different DBS sampling methods to each other (data not shown). Phe and Tyr concentrations measured in EDTA plasma were generally 7–12% higher compared to the other sampling types, except for LH DBS and EDTA DBS for Tyr.

## Discussion

To the best of our knowledge, our study is the first to report the potential differences between DBS and plasma Phe and Tyr concentrations in a wide concentration range, using data from both PKU and TT1 patients. Moreover, we investigated the differences between Phe and Tyr concentrations measured in different venous plasma samples and different DBS. Our results showed that differences, if any, are small with the applied analytical methods and DBS-plasma correction factor. To summarize our main findings: (1) no significant differences were observed between venous DBS and capillary collected DBS, (2) overall, LH plasma agreed well with the other ways of blood sampling, however when Phe concentrations are below approximately 50 μmol/L, i.e. low for PKU patients and usual for TT1 patients, differences with capillary DBS tended to appear (with Phe concentrations being lower in LH plasma), and (3) Phe and Tyr concentrations tended to be higher in EDTA plasma compared to other specimens.

Before discussing the results in more detail, some methodological issues are addressed. TT1 is a very rare inborn error of metabolism, also when compared to PKU. Only few TT1 patients could be included, and therefore our samples mostly derived from patients with high Phe and low/normal Tyr concentrations. The DBS-plasma correction factor therefore also mainly derived from samples of PKU patients, i.e. with low Tyr and high Phe concentrations. However, since both PKU and TT1 are being monitored by DBS, and using both groups gives the possibility to compare the full range of Phe and Tyr concentrations, we were interested in both patient groups.

Regarding the different ways of DBS sampling, our results showed no differences between capillary DBS, which are normally taken at home, and venous DBS, which are spotted at our outpatient clinic to enable Phe and Tyr measurement in DBS without needing an extra finger puncture in our patients. These results implicate that DBS sampled at home and at the outpatient clinic are considered to be comparable and can be used interchangeably. This finding has not been reported previously, but is important, because (1) most laboratories use DBS made of venous blood for validation purposes, (2) in our laboratory the DBS method is calibrated/corrected using DBS and plasma samples taken at the outpatient clinic and (3) if a DBS needs to be taken in the hospital alongside blood collection in tubes, it is both more efficient and patient-friendly to make a DBS from venous blood instead of capillary collected blood.

When comparing LH plasma to the other specimens, several (small) differences were observed, in particular for Phe concentrations. Interestingly, when we look at the agreement between the different methods by Bland Altman, a bias is especially observed in the lower Phe range with lower Phe concentrations in LH plasma when compared to capillary DBS. Particularly in this range, the differences between plasma and DBS are clinically relevant since low Phe concentrations have been associated with impaired growth, skin problems, and neurological deficits in both TT1 and PKU patients [5, 21]. For this reason, Phe supplementation is sometimes recommended in TT1 in case of persistently low Phe levels [4, 5, 7]. Furthermore, especially this lower range might be important with the upcoming new treatment for PKU patients, pegvaliase [22, 23]. Patients treated with pegvaliase often have low Phe concentrations, which can drop below the detection limit. The number of samples in this study with low Phe concentrations is however low and it should be noted that the observed bias may also be caused by the three outlying values. It is therefore unknown if this observed difference in the low Phe range is indeed real. Furthermore, the cause of this possible concentration-dependent difference remains unknown and further investigations with a higher number of samples with low Phe concentrations are needed.

The methods used to analyze plasma and DBS Phe concentrations both proved to be linear in the investigated concentration range, and concentrations were well above the limits of quantification. However, it is hypothesized that analytical challenges associated with DBS analyses could play a role causing high variability, including influences of hematocrit variation, chromatography effects on filter cards [24], and possibly inherent differences in Phe concentrations in capillary and venous blood. Factors associated with home blood sampling, in particular applied blood volumes, affecting e.g. spot volume per punch and extraction recoveries [25], were less likely to contribute because sampling occurred under controlled conditions at the outpatient clinic.

A surprising finding was that Phe and Tyr concentrations in EDTA plasma were 7–12% higher when compared to all other samples (Table 1). These differences have not been previously reported and so far, we have not been able to explain this difference. Since LH and EDTA plasmas of patients were analyzed in a single analytical run, analytical bias is not likely.

Several authors have investigated differences between plasma and DBS Phe and Tyr concentrations [9–12], but results are conflicting. Regarding Phe concentrations, only the study of Allard et al., who measured Phe and Tyr with both HPLC and flow-injection MS/MS (neonatal screening kit) methods, showed similar Phe concentrations in DBS and plasma [10]. All others reported lower Phe concentrations in DBS [9, 11, 12]. Stroup et al. reported significantly lower Phe concentrations in capillary DBS with a flow-injection MS/MS method and, to a lesser extent with ion exchange chromatography, compared to EDTA plasma [9]. Since we also found lower concentrations in DBS compared to EDTA plasma, it is possible that difference reported by Stroup et al. might, at least to some extent, be caused by the use of EDTA plasma rather than a structural difference between concentrations measured in plasma and in DBS. However, other studies also showed lower Phe concentrations in DBS compared to LH plasma [11, 12], indicating that other factors beside the used anti-coagulant cause the observed differences. One of these may be the difference between the analytical methods for DBS phe, i.e. flow-injection MS/MS analyses having lower DBS Phe concentrations compared to HPLC. This was also demonstrated by others [9, 10, 26], and may reflect differences in calibration and the applied extraction procedures. Reports on the Tyr concentration differences between plasma and DBS are scarce and inconclusive like with Phe. For instance, Allard et al. [10] reported no differences, and Groselj et al. [12] reported a 15.5% lower concentration in DBS.

Clinicians should be aware that large differences in DBS concentrations might exist between laboratories and between DBS and plasma, depending on the applied methods for calibration. Lack of standardization between methods impairs clinical decision making, since cut-off values for PKU and TT1 have been established to represent cut-offs for both plasma and DBS. Our results show that laboratories can achieve similar (mean) results of DBS and plasma, using DBS-plasma correction factors. Laboratories should however determine their own correction factor, since this is dependent on filter card types, and extraction and calibration protocols. Application of such a correction factor might generate better agreement between plasma and DBS samples, as is done regularly when using Point-Of-Care-Testing glucose meters. Standardization of the calibration procedure, i.e. application of lab-specific DBS-plasma correction factors, also improves the comparison of DBS and plasma Phe and Tyr concentrations within and between laboratories, which is essential in the development and adherence to guidelines for the monitoring of PKU and TT1 patients. To increase the inter-laboratory comparability, and therefore the applicability of both cut-off values from guidelines, the plasma method applied as gold standard, should be a method that is harmonized, preferably to international calibration standards, and be tested regularly in external quality control schemes, such as that provide by ERNDIM.

The large variability between DBS and plasma is more difficult to control. It has been shown that bias between plasma and DBS samples might be different among individuals [9]. Individually based differences may be caused by differences in hematocrit, home blood sampling techniques including applied blood volumes on the filter card, or unknown factors such as transport of the samples [9, 27, 28]. This was not investigated in this study since almost all patients were only included once and samples were taken in the outpatient clinic under controlled conditions. The high variability however, might be an explanation for the outliers that were observed.

Even though measurements of metabolites in DBS have (mainly) logistic advantages over measurements in plasma, the confounding pre-analytical factors invalidate the use of DBS for (home) monitoring. Regular education of patients on the DBS sampling procedure is essential to obtain reliable results. When DBS concentrations approach critical levels, a repeat measurement in plasma is advisable, if possible. In theory, monitoring using a Point-of-Care testing method for Phe and Tyr avoids the pre-analytical errors that are associated with DBS samples, and may not only decrease the total turn-around-time of the analyses, but may also result in lower variability and low bias, again when plasma-based calibrators are applied.

## Conclusions

In conclusion, using a DBS-plasma correction factor, our results show comparable Phe and Tyr concentrations in plasma and DBS. We recommend each laboratory to determine such a correction factor to improve the correlation between Phe and Tyr concentrations measured in venous blood and in DBS. Especially when relating metabolic control to clinical outcomes, it is important to keep in mind that there are differences between various blood sampling methods and that these might especially be dependent on the calibration method that is used.

## Supplementary information

**Additional file 1.** Supplemental Material. Detailed information of the method used for the determination of Phenylalanine and Tyrosine in dried blood spots.

## Data Availability

The datasets used and/or analyzed during the current study are available from the corresponding author on reasonable request.

## Abbreviations

DBS: Dried blood spots
EDTA: Ethylenediaminetetraacetic acid
LH: Lithium heparin
NTBC: 2-(2-nitro-4-trifluoromethylbenoyl)-1,3-cyclohexanedione
Phe: Phenylalanine
PKU: Phenylketonuria
TT1: Tyrosinemia type 1
Tyr: Tyrosine
