# Supplementary material for: Dried blood spot versus venous blood sampling for phenylalanine and tyrosine
Source: Orphanet J Rare Dis. 2020 Apr 3;15:82. doi: 10.1186/s13023-020-1343-7 (PMC7118958; doi:10.1186/s13023-020-1343-7)
Supplement: Supplementary file 1 — Additional file 1. Supplemental Material. Detailed information of the method used for the determination of Phenylalanine and Tyrosine in dried blood spots. [file 13023_2020_1343_MOESM1_ESM.docx]

**Supplemental Material**

**Detailed information of the method used for the determination of Phenylalanine and Tyrosine in dried blood spots**

Materials
Phenylalanine, Tyrosine, L-Phenyl-d5-alanine, and L-Tyrosine-(phenyl-d4) were obtained from Sigma-Aldrich Corporation (Saint Louis, Missouri, US). Methanol, Formic acid and HCl were obtained from Merck (Darmstadt, Germany). Blood spot cards (lot X124977) were obtained from Sartorius (TFN Grade 179 g/m^2^, Sartorius Stedim, Göttingen, Germany). These paper sheets are suitable for quantitative analyses of human fluids. A Quality Assurance Certificate was obtained, reporting weight (169-193 g/m2), thickness (0.40-0.49 mm), capillary rise lenghtwise 10 min (100-150 mm), water absorbance (205-368 g/m2), pH-value (6.0-7.0) and acidity (<0.06%).

Sample preparation
Phenylalanine (Phe) and Tyrosine (Tyr) calibration solutions in 0.1 N HCl (2.4 µl, range 25-1500 µm) were pipetted, and dried blood spot (DBS) punches (3.2 mm Ø) of samples and quality controls were placed in a 96-well plate. The samples were mixed with 150 µl internal standard solution (5 µM D5-Phe and 5 µM D4-Tyr in methanol). After 30 minutes of vortexing, the plate was centrifuged at 2000 rpm. 10 µl supernatant was transferred to a new 96-well plate and diluted with 190 µl 0,1% formic acid in MilliQ water. 10 µl of this sample was injected onto the column.

Analytical method

DBS Phe and Tyr concentrations were measured using high-performance liquid chromatography (LC20; Shimadzu, Kyoto, Japan) coupled to a triple quadruple mass spectrometer with an electrospray ionization source (API-3200, SCIEX, Framingham, MA, USA). Liquid chromatography tandem mass spectrometry (LC-MS/MS) analysis was carried out using a Kinetex Biphenyl (2.6 µm pore size, 150 x 4.6 mm) analytical column coupled to a Kinetex Biphenyl (2.6 µm pore size, 2 x 4.6 mm) guard column (Phenomenex, Torrance, California, USA). Separation was achieved in 4 minutes applying isocratic elution [50% mobile phase A: 0.1% formic acid in MilliQ water and 50% mobile phase B: 100% methanol], at a flow rate of 0.6 mL/min. Detection was achieved using positive-ion electrospray ionization in multiple reaction monitoring mode, using the following transitions: m/z 171.1🡪125.1 for D5-Phe, 166.1🡪120.2 for Phe, 186.1🡪140.3 for D4-Tyr and 182.1🡪136.2 for Tyr. The electrospray ionization source temperature was kept at 450 ^o^C, the ion spray voltage at 4000V and nitrogen was used as nebulizing gas. Data were analyzed using Analyst 1.6.2 (Sciex). The blood spot Phe + Tyr method was linear up to 3000 µM. Limits of detection (LOD) and limits of quantification (LOQ) were determined based on the signal-to-noise ratio approach, using ratios of three and ten respectively. The LOD/LOQ were 1.0/2.0 µM for DBS Phe and 1.0/5.0 µM for DBS Tyr. Recoveries in DBS from whole blood samples of an apparently healthy adult spiked with 100-1000 µM Phe and Tyr were 87.5-103.7% and 87.3-101.6%, respectively. Variation coefficients of the internal quality control samples of the DBS LC-MS/MS method and the plasma Biochrom method during the study period are shown in Supplemental Table 1.

Calibration

Calibration solutions in 0.1N HCl were used instead of blood spot calibrators, the latter being the gold standard for analysis of metabolite concentrations in blood spots. These solutions have advantage over blood spots given the higher analytical precision resulting in lower inter-assay variability. It was possible to use these solutions because concentrations of Phe and Tyr in DBS were calculated from the concentrations in plasma using a DBS-plasma correction factor. The correction factor was determined by constructing a calibration curve in 0.1N HCl and by spiking whole blood samples of an apparently healthy male adult with Phe and Tyr (0-100-250-500-750-1000-1400 µmol/l). One drop of blood was spotted and dried for at least 3 hours on filter paper, and corresponding plasma was prepared by centrifugation of the remaining blood. Phe and Tyr were analysed in duplicate in 3.2 mm Ø punches with LC-MS/MS, and calculated based on the calibration curve in 0.1N HCl (using 1 µl of calibration standard). This can be considered the first calibration step. The spiked plasma samples were subsequently analysed in duplicate with our amino acid reference method (Biochrom, see main article). The DBS-plasma correction factor was estimated by means of a Passing and Bablok regression analysis, by plotting the Phe or Tyr concentrations in plasma on the x-axis (Biochrom method) and the concentrations in blood spots on the y-axis (LC-MS method). The slope of this regression analyses corresponds to the DBS-plasma correction factor. This factor can be considered the second calibration step. The regression analyses demonstrated a slope of 2.4. As this correction factor has been determined several years ago and the lab information system has been replaced, the original data that generated this correction factor cannot be shown. To illustrate the origin of the correction factor, Figure 1 has been added, presenting a theoretical figure of the Passing and Bablok analysis that was used to determine this correction factor of 2.4. We apply this correction factor in the amount of calibration solution (combining the first and second calibration steps). This corrects blood spot Phe and Tyr to their respective plasma concentrations. It should be realized that this factor only applies to the given extraction conditions and filter cards. The factor was verified by comparing Phe and Tyr concentrations in lithium heparin plasma with Phe and Tyr concentrations in blood spots from venous blood (the latter obtained via syringe) of 13 patients visiting our outpatient clinic, using Passing and Bablok regression analyses, as applied in the main manuscript. This analysis revealed no significant differences. and the results of the current study also verifies the accuracy of the correction factor.

**Supplemental Table 1.** Internal quality control results during the study period.

|  | Biochrom 30  Plasma | Biochrom 30+  Plasma | LC-MS/MS  Dried blood spot |
| --- | --- | --- | --- |
| **Phenylalanine**  Level 1  *CV*  *Mean±sd*  Level 2  *CV*  *Mean±sd* | Lot 1 / Lot 2  3.9% / 3.5%  (342.2±13.3 / 362.4±12.8)  3.9%  (665.9±25.9) | Lot 1 / Lot 2  4.3% / 3.7%  (342.5±14.3 / 358.9±13.4)  3.0%  (671.7±20.5) | Lot 1 / Lot 2  9.6% / 7.2%  (107.0±10.3 / 145±10.5)  7.4% / 6.9%  (886.0±65.5 / 926.3±63.6) |
| **Tyrosine**  Level 1  *CV*  *Mean±sd*  Level 2  *CV*  *Mean±sd* | Lot 1 / Lot 2  4.2% / 3.4%  (232.0 ± 9.7 / 82.9 ± 2.8)  3.3%  (888.2 ± 29.4) | Lot 1 / Lot 2  3.6% / 3.5%  (231.3 ± 8.4 / 84.0 ± 2.9)  2.9%  (897.3 ± 24.5) | Lot 1 / Lot 2  10.0% / 8.2%  (96.9 ± 9.7 / 123.3 ± 10.1)  9.1% / 7.8%  (703.7 ± 61.3 / 751.6 ± 58.4) |

Data represent variation coefficients (CV%) and mean±sd (µmol/l) for the internal QC batch samples. Two lots of QC samples were analyzed during the study period, except for the amino acids QC level 2.

*
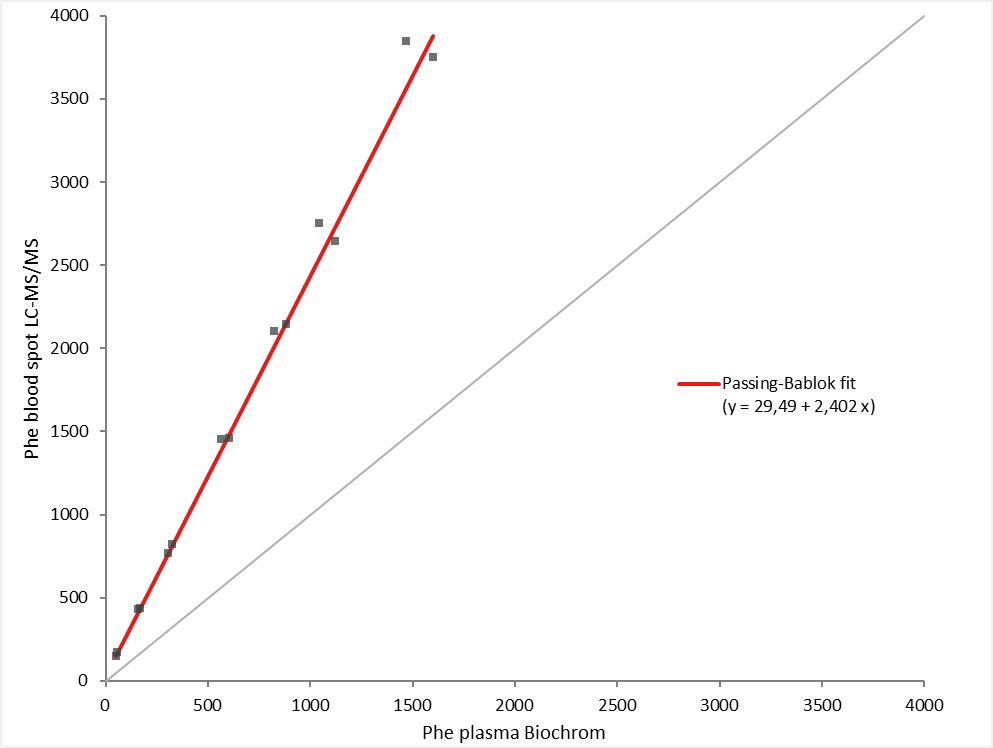
*

**Supplemental Figure 1.** Passing and Bablok regression analysis (theoretical model). Phe (or Tyr) concentrations were plotted of samples analysed in duplicate with Biochrom in plasma, and with LC-MS/MS in dried blood spot using 1 µl of calibration solution for the latter method. Passing and Bablok analyses show their relation (plasma on x-axis, dried blood spot on y-axis). This generated a slope of 2.4 in this example and this should be used as the correction factor. Note the intercept of 29.5, which is the endogenous concentration of Phe in the whole blood sample, which should be ignored for the correction factor.
